# The efficacy and safety of traditional Chinese medicine physiotherapy combined with acupoint injection on diabetic peripheral neuropathy: A protocol for systematic review and meta-analysis

**DOI:** 10.1097/MD.0000000000031467

**Published:** 2022-12-16

**Authors:** Lin Wang, Bugao Guan, Guangrun Li, Liquan Feng, Hui Sun, Jin Xu

**Affiliations:** a Department of Rehabilitation Medicine, Jinhu County People’s Hospital, Huai’an, Jiangsu Province, China.

**Keywords:** Acupoint injection, diabetic peripheral neuropathy, protocol, Traditional Chinese medicine physiotherapy

## Abstract

The efficacy and safety of traditional Chinese medicine physiotherapy combined with acupoint injection in treating diabetic peripheral neuropathy remains unknown. As a result, we will conduct a systematic review and meta-analysis to assess the evidence.

**Methods:**

We will look for pertinent randomized controlled trials in the following databases: China National Knowledge Infrastructure, WanFangData, Chinese biological medical database, Medline, Cochrane Library, PubMed, and Embase up to January 2022. Following the standards of Cochrane Review 6.2, 2 researchers independently evaluated the quality of the evidence in the relevant papers. Data analysis will be conducted by using Review Manager 5.4, including statistical analysis, subgroup analysis, making forest plot and funnel chart.

**Results:**

The results will be submitted to a peer-reviewed journal.

**Conclusion:**

The research will verify the safety and efficacy of traditional Chinese medicine physiotherapy in combination with acupoint injection for diabetic peripheral neuropathy.

## 1. Introduction

Diabetes mellitus is a metabolic disease characterized by hyperglycemia caused by various causes, which increases significantly in recent years.^[1–5]^The prevalence of diabetes in China was 11.2%.^[6]^

The number of diabetes patients in China ranks first in the world.^[7,8]^ Diabetic peripheral neuropathy (DPN) is a common microvascular complication of diabetes that affects a lot of people. It usually causes pain, numbness or tingling, symmetrical “glove or sock-type” sensory disturbances in the limbs, and it gets worse at night or when it’s cold.^[9]^ Diabetes-related peripheral neuropathy can have serious consequences, including amputation and ulcers.^[10]^ Diabetes duration,^[11,12]^ age,^[11–14]^ HbA1c,^[15,16]^ smoking,^[17]^ body mass index, and fasting plasma glucose^[18,19]^ are all risk factors for DPN.

Nearly half of people with diabetes have gradually developed DPN.^[20–22]^ Statistics show that 30%, 60%, and 90% of people with diabetes after 5, 10, and 20 years get DPN, which has a big impact on their quality of life.^[23]^

Clinically, Western medicine methods for treating DPN mainly concern blood sugar control,^[24]^ drug therapy, neurolysis,^[25]^ and electrical nerve stimulation.^[26,27]^ In contrast, traditional Chinese medicine methods are mainly acupuncture, moxibustion, and Chinese oral medicine.^[28–30]^

Therefore, a safer and more effective treatment method is urgently needed. Traditional Chinese medicine physiotherapy refers to a therapy that promotes local and systemic blood and lymph circulation and improves local nutrition and systemic functions through the action of heat and drugs. Acupoint injection is a type of treatment that involves choosing certain acupoints and using a syringe to take out a small amount of a drug to inject into those points.

In recent years, an increasing number of studies have reported the combined use of the above 2 therapies in the treatment of DPN, but their safety and efficacy are still unclear. The objective of this study is to verify the clinical efficacy and safety of traditional Chinese medicine physiotherapy combined with acupoint injection in the treatment of DPN.

## 2. Methods

### 2.1. Study registration

This study has been registered on the PROSPERO. The registration number is CRD42022358413. The method used for this protocol will follow Preferred reporting items for systematic review and meta-analysis protocols (PRISMA-P) statement.

### 2.2. Eligibility criteria

#### 2.2.1. Types of studies.

The types of included studies are randomized controlled trials (RCT) of the efficacy and safety of traditional Chinese medicine physiotherapy and acupoint injection for DPN. Language is limited to English and Chinese. We will exclude them if the types of articles are cases, reviews, abstracts, and comments.

#### 2.2.2. Types of participants.

We will include patients who have been diagnosed with DPN.

#### 2.2.3. Types of interventions.

We will consider using both traditional Chinese medicine physiotherapy and acupoint injection as the experimental group.

#### 2.2.4. Types of comparators.

We will consider other treatments as the control group, except traditional Chinese medicine physiotherapy and acupoint injection.

#### 2.2.5. Types of outcome measures.

We will consider the following methods as the outcome measures: Efficacy, clinical symptom score, and nerve conduction velocity.

We will take clinical efficacy as the primary outcome, nerve function will be considered as secondary outcome.

## 3. Exclusion criteria

1. Repeated published literature.
2. Analysis of missing data.
3. Retrospective literature.
4. Non-Chinese and English literature.
5. Literature with no needed outcomes in the literature.
6. Literature with unqualified trial design (for example, no explicit trial control in the trial).
7. Non-RCTs
8. Animal trials.

### 3.1. Information sources

The China National Knowledge Infrastructure, WanFangData, Chinese biological medical database, Medline, Cochrane Library, PubMed, and Embase databases will be searched to collect the RCT studies about the efficacy and safety of traditional Chinese medicine physiotherapy combined with acupoint injection for DPN up to January 1, 2022.

### 3.2. Search strategy

We will search different databases for eligible literature. The search terms will include the following terms: RCT, diabetic Neuropathy, peripheral neuropathy, traditional Chinese medicine physiotherapy, acupoint injection, and their Medical Subject Headings. The Search strategy will be offered to peer-review is Table 1.

**Table 1:** The search strategy will be used in the PubMed database.

Search number	Search terms
1	Randomized controlled trial
2	Controlled clinical trials
3	Randomly
4	Randomized
5	Trial
6	Diabetic Neuropathies
7	Diabetic Neuropathy
8	Peripheral
9	Peripheral Nervous System Diseases
10	Peripheral Neuropathies
11	Peripheral Neuropathy
13	1 OR 2-5
14	6 OR 7
15	8 OR 9-11
16	Medicine, Chinese Traditional
17	Traditional Chinese Medicine
18	Meridians
19	Acupuncture Points
20	Acupuncture Point
21	Acupoints
22	Acupoint
23	Injections
24	Injection
25	16 OR 17
26	18 OR 19-22
27	23 OR 24
28	13 AND 14 AND 15 AND 25 AND 26 AND 27

### 3.3. Data management

The basic information and data will be managed by 2 independent researchers. We will use Endnote X9 to manage eligible literature and make use of Microsoft Excel to make tables for the basic information and study statistic data.

### 3.4. Selection process

Two independent researchers will be assigned to identify qualifying studies. To begin, we will scour the databases for relevant literature by reading the title and abstract. Then we will get the whole text of the remaining literature and read it. Finally, after removing all of the literature using exclusion criteria, the remaining literature will be included in the systematic review and meta-analysis. If there is a disagreement about the selection of literature, the third researcher will enter the conversation and settle it.

### 3.5. Data collection process

Two researchers will be assigned to gather data. First, we will gather qualifying information in Microsoft Excel. If the findings are contentious, the third researcher will engage in the debate and solution. The data will be extracted using the pre-created table.

### 3.6. Data items

The initial author’s name, the sample size, the publication date, the age of the patients, the gender of the participants, the treatment period, the length, the outcome indicators, the adverse events, and frequency will all be included in the basic information.

If the original study data description in the literature is unclear or there is a paucity of data, we will contact the author through email to get it.

### 3.7. Study risk of bias assessment

Two independent researchers will conduct the risk of bias assessment. The quality evaluation criteria recommended by Cochrane Review include random sequence generation (selection bias), allocation concealment (selection bias), blinding of participants and personnel (performance bias), blinding of outcome assessment (detection bias), incomplete outcome data (attrition bias), selective reporting (reporting bias), and other bias.

We will determine the judgments of “yes” (low risk), “no” (high risk), and “unclear” (lack of relevant information or uncertainty of bias) for each literature.

### 3.8. Statistical analysis

Two researchers will conduct a meta-analysis by Review Manager 5.4 independently. The combined mean difference with 95% confidence intervals will be used for the continuous data and the combined odds ratio and relative risk with 95% confidence interval will be used for the dichotomous data.

The Chi-square test will be used to evaluate the statistical heterogeneity among the results of the studies. The size of heterogeneity will be quantitatively judged by *I*^*2*^. When the test results are *P* > .05 and *I*^*2*^ < 50%, the fixed effect model will be used for meta-analysis. If the test results are *P* < .05 and *I*^*2*^ > 50%, we will analyze the recourses of the heterogeneity and choose the random effect model.

Subgroup analyses will be carried out on the assumption that the heterogeneity is significant.

### 3.9. Sensitivity analysis

We will exclude each study to judge the resource of the heterogeneity if necessary.

### 3.10. Publication bias

If there is an outcome that includes 10 or more studies, we will make a funnel chart for publication bias assessment.

### 3.11. Confidence in cumulative evidence

We will use the Grades of Recommendations Assessment Development and Evaluation approach to verify the quality of evidence of the included studies. The Grades of Recommendations Assessment Development and Evaluation approach divides the quality of evidence into very low, low, moderate, or high.

### 3.12. Ethics approval

The study does not require ethical approval because of failing to involve the patients.

## 4. Discussion

The research aims to verify the efficacy and safety of traditional Chinese medicine physiotherapy and acupoint injection for the clinic. Traditional Chinese medicine physiotherapy and acupoint injection are prevailing methods that are used increasingly for DPN in recent years. However, there is no meta-analysis and systematic review concerning their safety and efficacy of them. So, it is necessary to testify to their safety and efficacy of them if they can be used as an alternative to the traditional treatment.

## Acknowledgments

The authors would like to express their gratitude to the Huai’an Commission of Health.

## Author contributions

**Conceptualization:** Lin Wang,

**Data curation:** Lin Wang, Bugao Guan.

**Formal analysis:** Bugao Guan, Jin Xu.

**Funding acquisition:** Lin Wang.

**Methodology:** Guangrun Li, Lin Wang.

**Resources:** Guangrun Li, Lin Wang.

**Software:** Liquan Feng, Jin Xu.

**Writing – —original draft:** Liquan Feng, Jin Xu.

**Writing – —review & editing:** Hui Sun, Jin Xu.
